# Supplementary material for: Enhanced YOLOv8-based pavement crack detection: A high-precision approach
Source: PLoS One. 2025 May 22;20(5):e0324512. doi: 10.1371/journal.pone.0324512 (PMC12097708; doi:10.1371/journal.pone.0324512)
Supplement: Attachment — Submitted filename: Response to Reviewers.docx [file pone.0324512.s002.docx]

## Response to reviewers

Dear editor and reviewers,

Thank you for offering us an opportunity to improve the quality of our submitted manuscript (Enhanced YOLOv8-Based Pavement Crack Detection: A High-Precision Approach). We appreciated very much the reviewers' constructive and insightful comments. In this revision, we have addressed all of these comments. We hope the revised manuscript has now met the publication standard of your journal.

We highlighted all the revisions in red colour. On the next pages, our point-to-point responses to the queries raised by the reviewers are listed.

**Response to Reviewer #1:**

**Q1: The manuscript should provide a more detailed explanation of the YOLOv8-DGS model improvements. Specifically, elaborating on how deep separable convolution (DWConv) and GSConv are integrated into the backbone and neck of the model would help readers understand the technical advancements made in crack detection.**

A1: Thank you very much for your suggestions, which are very important to improve the quality of the paper. we describe the model improvements in detail in Section 3.2, further explaining how and why the modules are integrated. Your suggestions make our article more complete. Changes have been highlighted in red.

**Q2: These references could be cited in the introduction, particularly when discussing the advancements in deep learning methods for image processing and detection tasks. It would fit well in a paragraph that highlights the growing application of deep learning in various fields, including remote sensing and crack detection. Remote Sensing, 15(10), 2663. [https://doi.org/10.3390/rs15102663,](https://doi.org/10.3390/rs15102663,) Ocean Engineering, 259, 111735. [https://doi.org/10.1016/j.oceaneng.2022.111735](https://doi.org/10.1016/j.oceaneng.2022.111735).**

A2: Thank you for your valuable comments. We believe that the references you provided are more suitable for this article to discuss the current development of deep learning. The revised citations have been marked in red.

**Q3: This reference can be cited in the introduction, particularly when discussing advancements in the YOLOv8 model, machine learning, and its applications in various fields, including marine debris detection. It would be relevant to mention this study when highlighting the versatility and improvements of YOLOv8 in different contexts. Journal of Marine Science and Engineering, 12(10), 1748. [https://doi.org/10.3390/jmse12101748,](https://doi.org/10.3390/jmse12101748,) International Journal of Computational Methods, 2450066. 10.1142/S021987622450066X, Journal of Computing in Civil Engineering, 39(3), 4025017. 10.1061/JCCEE5.CPENG-6167, Scientific Reports, 14(1), 15170. 10.1038/s41598-024-66234-3, Measurement Science and Technology, 36(1), 015104. 10.1088/1361-6501/ad7f77, GPS Solutions, 28(4), 178. 10.1007/s10291-024-01715-6, IEEE Transactions on Vehicular Technology, 1-16. 10.1109/TVT.2024.3492388.**

A3: Thanks for your valuable comments. After careful comparison, we found that most of the documents you provided were more suitable than our original references, and made corresponding modifications after summarizing them. The modified parts have been highlighted in red.

**Q4: It is essential to include a comprehensive description of the experimental setup used to evaluate the YOLOv8-DGS model. This should encompass the dataset characteristics, the sample number, and the test case selection criteria. Providing this information will enhance the reproducibility of the study.**

A4: Thank you for your valuable advice. In order to improve the repeatability of the study, we modified the content of the part of materials and methods, and described the size and resolution of the data set and the reason for the division of the data set in detail. In the experimental part, the machine configuration and training parameters are written out in detail. Your suggestions add value to our research.

**Q5: While the manuscript mentions impressive performance metrics such as Precision, Recall, F1-score, and mAP50, it would be beneficial to include a comparison with existing models, such as YOLOv3 or other state-of-the-art methods. This comparison can highlight the advantages of the proposed model and provide context for the reported results.**

A5: Thank you very much for your valuable suggestions, which are very important to improve the quality of the paper. Adding the content of comparison with the current advanced models in the abstract can better highlight the research results. We have added the comparison with the experimental results of yolo11 according to your suggestions.

**Q6: These references could be cited when discussing the importance of real-time identification systems in various environments, including underground disaster areas. It would be relevant to mention this study while highlighting the advancements in detection technologies and their applications in safety and rescue operations. A potential sentence could be: "Recent studies have demonstrated the effectiveness of multi-source heterogeneous data fusion in real-time identification of critical situations in disaster areas, which aligns with the advancements in detection technologies discussed in this paper. Safety Science, 181, 106690. [https://doi.org/10.1016/j.ssci.2024.106690.](https://doi.org/10.1016/j.ssci.2024.106690.) Remote Sensing, 15(17), 4251. [https://doi.org/10.3390/rs15174251.](https://doi.org/10.3390/rs15174251.) Advances in Civil Engineering, 2023(1), 8897139. [https://doi.org/10.1155/2023/8897139.](https://doi.org/10.1155/2023/8897139.) Chinese Journal of Mechanical Engineering, 37(1), 108. 10.1186/s10033-024-01107-4. Computers and Geotechnics, 178, 106949. [https://doi.org/10.1016/j.compgeo.2024.106949.](https://doi.org/10.1016/j.compgeo.2024.106949.) Computers and Geotechnics, 177, 106827. [https://doi.org/10.1016/j.compgeo.2024.106827](https://doi.org/10.1016/j.compgeo.2024.106827).**

A6: Thank you for your valuable comments. After reviewing the original literature and speech, we found that there are some problems. Your suggestions can help us optimize this part. It has now been modified, with the modified parts marked in red.

**Q7: Incorporating visual aids, such as graphs or tables, to present the performance metrics and comparisons with other models can significantly improve the manuscript's clarity and impact. Visual representations can help readers quickly grasp the improvements made by the YOLOv8-DGS model.**

A7: Thanks for your valuable suggestions. Visualization tools can help us express more intuitively. We have added some tables and heat maps to reflect the model training effect before and after image processing.

**Q8: The manuscript should address any limitations of the proposed method. Discussing potential challenges, such as the model's performance in varying environmental conditions or with different types of pavement materials, would provide a balanced view of the research.**

A8: Thanks for your comments, we think it is necessary to write out the limitations of the model. According to your opinions, we have added the existing problems and challenges faced by the improved model, and your valuable suggestions have made our conclusion more complete.

**Q9: Including a section on future work could enhance the manuscript. Suggestions for further research, such as exploring the application of the YOLOv8-DGS model in real-time scenarios or integrating it with other technologies, would demonstrate the ongoing relevance of the research.**

A9: Thank you for your valuable advice, the content about future work is indispensable. We supplement this part by summarizing the current shortcomings and further work of the research. Your suggestions make our articles more orderly.

**Q10: A thorough manuscript proofreading is recommended to ensure clarity and coherence. This includes checking for grammatical errors, ensuring consistent terminology, and improving the overall flow of the text.**

A10: Thanks for your valuable advice, we have proofread the manuscript thoroughly and corrected the grammatical errors to make the article smooth and smooth. Thank you again for your valuable comments, and we hope the revised paper can be approved by you.

**Response to Reviewer #2:**

**Q1: The title is clear and informative but could be slightly refined. Suggested improvement: "Enhanced YOLOv8-Based Pavement Crack Detection: A High-Precision Approach". Consider explicitly mentioning "Deep Learning" or "Computer Vision" to attract a broader audience.**

A1: Thank you very much for your valuable suggestions, which are very important to improve the quality of the paper. Compared with the previous title, the title you suggested is more attractive. I have modified the title, and the modified part is marked in red.

**Q2: In the abstract section add a sentence highlighting how this method outperforms conventional YOLO models. The section lacks a direct comparison with existing YOLO models. It also does not explicitly mention real-time feasibility or computational efficiency. The claim about "improving performance" is general—what aspect? Accuracy, speed, or robustness? Specify if this model is suitable for real-time deployment.**

A2:Thank you for your valuable comments. We did have some missing information in the summary, detailed the improvement in recognition accuracy, compared with the improved model, and indicated the running speed of the model. Your suggestions made our article more clear and easy to understand.

**Q3: In the introduction section, the paper mentions manual inspection is inefficient but does not provide statistics or real-world implications (e.g., cost/time of manual inspection vs. AI-based detection). The problem statement could be more focused. Instead of stating that "YOLOv8 has potential," explicitly highlight its limitations in pavement crack detection. The introduction does not clearly state YOLOv8 alone is insufficient. Instead of saying "YOLOv8 has potential," it should specify its weaknesses (e.g., handling small cracks, computational load). Other crack detection methods (e.g., transformer-based approaches) are missing. How does this work compare to recent works like CrackFormer? The paper should explicitly state the gap in previous research that this study addresses.**

A3: Thanks for your valuable comments, which helped us a lot in the introduction section of our article, we thought it would be more helpful to provide statistical data on the current situation analysis, we highlighted its limitations and weaknesses in introducing yolov8, and explained the disadvantages of the recent CrackFormer model compared to yolo. Make the article more reasonable by modifying the parts you suggested. The modified parts have been highlighted in red.

**Q4: In the material and method section, the sample size and its justification need more emphasis. A dataset split ratio (80:20) is mentioned, but no rationale is provided. Does the dataset cover diverse road conditions (e.g., lighting, shadows, different pavement materials)? What resolution are the images? Are there any preprocessing steps to remove blur/noise? Will a model trained on RDD2022 generalize well to different environments? The section could benefit from a statistical analysis of how preprocessing improved accuracy.**

A4: Thanks for your valuable suggestions, we found that the article does lack the detailed description of the data set and the reasons for the proportion division of the training set and test set. Now we have revised this part, adding image resolution, road conditions, etc., and focusing on the description of the reasons for the division of the data set. Your suggestions make our Materials and methods chapter more complete. Changes have been highlighted in red.

**Q5: Section 2.2… How does preprocessing affect accuracy? A small table comparing performance before/after preprocessing would strengthen this section. Mean filtering is explained but could use more mathematical rigor. Why was mean filtering chosen? Does it outperform Gaussian filtering or median filtering for noise reduction?**

A5: Thanks to your valuable advice, we have created a table that clearly and intuitively highlights the importance of preprocessing and adds the formulas used for filtering equalization operations. Gaussian filtering is suitable for uniform noise removal, but will blur the edges. Median filtering is good for salt-and-pepper noise, but it may lose the detail information of cracks. Bilateral filtering not only smoothes the noise, but also preserves the feature of crack edge better, so it is most suitable for this application scenario.

**Q6: Section 3… Detection algorithm improvement… The paper claims the improvements reduce computational load, but where is the evidence? A table comparing FLOPs and parameter count before and after modification is needed. How do the modifications affect feature extraction? A grad-CAM visualization would strengthen this section.**

A6: Thanks for your valuable suggestions, adding a visual performance improvement can make our research more intuitive. We have added a table in the corresponding section to highlight the reduction in the calculation of the improved model, and added a heat map to show the improvement in the model's physical signs extraction capability. Your advice was very valuable and we thank you again.

**Q7: In the experimental section of the paper, were hyperparameters optimized via grid search? Why were specific values chosen? Would adding more layers improve accuracy?**

A7: In this study, the hyperparameters of the model are not globally optimized by grid search, but adjusted by empirical tuning and random search. Grid search usually traverses all possible parameter combinations in hyperparameter optimization. Although it can find the global optimal solution, it has a high computational cost, especially in the training process of deep learning models, which requires a lot of computational resources. Therefore, we adopted a more efficient random search, randomly sampled multiple combinations within a reasonable parameter range for the experiment, combined with the convergence rate of the model, the change trend of loss function, mAP curve and other indicators to select the optimal hyperparameter. Whether increasing the number of network layers improves accuracy depends on a number of factors, including the complexity of the data set, the computational power of the model, and whether a suitable regularization mechanism is introduced. In this study, the number of network layers of YOLOV8-DGS was slightly adjusted compared with standard YOLOv8 (from 168 layers to 174 layers), with the main purpose of optimizing feature extraction and fusion, rather than blindly increasing the number of layers. Although increasing the number of layers can enhance the feature expression ability of the model and improve the detection ability of complex fracture morphology. But at the same time, it will also lead to an increase in the amount of computation and a decrease in the reasoning speed, which is not conducive to the real-time detection needs of the intelligent sewing machine. If the amount of data is insufficient, the network that is too deep is prone to overfitting, resulting in a decline in generalization ability. Therefore, there is no fixed conclusion on whether increasing the number of layers can improve the accuracy. When improving YOLOv8, this study is more inclined to improve the detection performance of the model by optimizing the network structure, adjusting the feature extraction method, and improving the data enhancement strategy, rather than simply increasing the number of layers.

**Q8: Section 4.2…. Are differences statistically significant? A p-value analysis is needed. A statistical test comparing YOLOv8-DGS with standard YOLO models would strengthen claims. "Inference Time" should be broken down into GPU vs CPU performance.**

A8: Thanks for your valuable suggestions, we believe that adding P-value analysis can well verify the effectiveness of the improved model, and your suggestions make our paper more rigorous. In Section 4.1 of this paper, we describe the experimental platform of this research in detail. Experiments show that the inference speed of gpu is much higher than that of cpu, and the gpu platform is more suitable for deep learning.

**Q9: Section 5… Conclusion section lacks a discussion of failure cases: When does the model fail? Low contrast? Blurry images? Future work is vague: What specific lightweight techniques will be explored? No discussion of practical deployment: How can this be integrated into real-world applications?**

A9: Thanks for your valuable comments, it is important to research current issues and future work, and we have added these missing pieces: current shortcomings of the model, current hot lightweight technologies, and future deployment efforts. Your suggestions make the conclusion of our article more complete. Thank you again for your valuable comments, and we hope the revised paper can be approved by you.

**Response to Reviewer #3:**

**Q1: How do you measure the “accuracy” in the case study? It is the most important criterion. Its result is given without calculation or definition. How do you know the actual measurement of the cracks?**

A1: Thank you for your recognition and suggestion to accept my paper. We believe that accuracy is indeed too general, and we have revised it according to your questions. Accuracy refers to the values of various evaluation indicators obtained from improved model training, such as Precision, Recall, F1-score, mAP50 and FPS, which reflect the accuracy and detection speed of the model. The result data in the experimental part reflects the recognition accuracy of the model in the actual detection. The calculation of crack size is related to the research of image segmentation in the future, and we will solve this problem in the following research.

**Q2: Compared to YOLO 11, YOLOv8-DGS has better accuracy on mAP50 but worse accuracy on MAP50-95. Their running speeds are also similar. Does it mean YOLOv8-DGS is not better than YOLO 11? Please explain.**

A2: Thanks for your valuable comments, the experimental results in the experimental part of our article show that the improved YOLOv8 model is better than yolo11 in mAP50-95 and running speed.

**Q3: This paper has many typos and errors, for example, it should be Table 3 instead of Figure 3 on Line 354. Please carefully revise this paper before publication.**

A3: Thanks for your valuable suggestions, we have carefully corrected the spelling mistakes, and carefully checked the graph corresponding to the text, your suggestions have made our article more rigorous. Thank you again for your valuable comments, and we hope the revised paper can be approved by you.
